# The Membrane-Bound Notch Regulator Mnr Supports Notch Cleavage and Signaling Activity in *Drosophila melanogaster*

**DOI:** 10.3390/biom11111672

**Published:** 2021-11-10

**Authors:** Anja C. Nagel, Dominik Müller, Mirjam Zimmermann, Anette Preiss

**Affiliations:** Department of General Genetics 190g, University of Hohenheim, Garbenstr. 30, 70599 Stuttgart, Germany; muelled@gmx.de (D.M.); zimmermann.mirjam@uni-hohenheim.de (M.Z.); a.preiss@uni-hohenheim.de (A.P.)

**Keywords:** cell–cell communication, Notch cleavage, γ-secretase, membrane-bound Notch regulator, Mnr, Notch, signaling dynamics, intramembrane proteolysis

## Abstract

The Notch signaling pathway is pivotal to cellular differentiation. Activation of this pathway involves proteolysis of the Notch receptor and the release of the biologically active Notch intracellular domain, acting as a transcriptional co-activator of Notch target genes. While the regulation of Notch signaling dynamics at the level of ligand–receptor interaction, endocytosis, and transcriptional regulation has been well studied, little is known about factors influencing Notch cleavage. We identified EP555 as a suppressor of the Notch antagonist Hairless (H). EP555 drives expression of CG32521 encoding membrane-bound proteins, which we accordingly rename *membrane-bound Notch regulator* (*mnr*). Within the signal-receiving cell, upregulation of Mnr stimulates Notch receptor activation, whereas a knockdown reduces it, without apparent influence on ligand–receptor interaction. We provide evidence that Mnr plays a role in γ-secretase-mediated intramembrane cleavage of the Notch receptor. As revealed by a fly-eye-based reporter system, γ-secretase activity is stimulated by the overexpression of Mnr, and is inhibited by its knockdown. We conclude that Mnr proteins support Notch signaling activity by fostering the cleavage of the Notch receptor. With Mnr, we identified a membrane-bound factor directly augmenting Notch intra-membrane processing, thereby acting as a positive regulator of Notch signaling activity.

## 1. Introduction

Cell–cell communication is a fundamental principle for the specification and differentiation of the diverse cell types that build a multicellular animal. The Notch signaling pathway is one of several highly conserved signaling pathways mediating intercellular communication. Accordingly, Notch signals are pivotal to normal development, and alterations are associated with various congenital diseases, as well as cancer formation (reviewed in [1,2]). Hence, understanding the principles underlying the regulation of this pathway is of great interest. An extraordinarily complex network of genes, mostly identified in *Drosophila melanogaster*, affect Notch signaling activity, but many remain to be characterized in detail (reviewed in [3]).

Notch earned its name through a dominant mutation in *Drosophila*, on the basis of the wing incisions displayed by adult heterozygous flies [4]. Much later, it was recognized that the Notch pathway is specifically activated in the abutting cells of the dorsal and ventral compartments within the wing anlagen. At this interface, Notch activity establishes a growth organizer, and eventually the wing margin is formed. Incomplete Notch signaling activity causes gaps in the boundary, and hence, wing notches arise (reviewed in [5]). Notch signaling can only take place between neighboring cells in direct contact, as both the Notch receptor and its ligands are transmembrane proteins. There are two classes of Notch ligands, named Delta and Jagged in vertebrates, and Serrate in flies, respectively. The signaling cell presents the ligand on its surface; the signal-receiving cell bears the Notch receptor. Upon ligand binding, the Notch receptor is cleaved within the transmembrane domain to release its intracellular domain NICD. Acting as a co-activator in multi-protein complexes, NICD then induces the transcriptional activation of Notch target genes, thereby translating receptor activation into specific cellular responses (reviewed in [5,6]). Transcriptional regulation of Notch target genes relies on the DNA-binding molecule CSL (acronym for CBF-1 in mammals, Suppressor of Hairless Su(H) in *Drosophila*, and Lag-1 in *Caenorhabditis*) acting as a molecular switch. By recruiting particular cofactors, CSL builds up activator and repressor complexes, resulting in gene activation and repression, respectively (reviewed in [6]).

At first glance, the Notch signaling pathway appears simple and direct, lacking distinct amplification steps, for example, by phosphorylation, that could serve as adjustment factors. Instead, regulation occurs primarily at the level of receptor–ligand interactions, the internalization of the Notch receptor, and at the transcriptional level (reviewed in [2,5,7]). The binding of the ligand to the Notch receptor is largely influenced by post-translational modifications, primarily by glycosylation, of either one (reviewed in [8,9,10]). Yet, none of these modifications has been shown to directly influence the cleavage of the Notch receptor, i.e., the release of the biologically active NICD fragment. Instead, a mechanical pulling force, administered by the signaling cell via ligand internalization, unfolds an extracellular cleavage site for ADAM metalloproteases within the Notch receptor, termed the S2 site. S2 cleavage is a premise for the intramembrane proteolysis of Notch by a presenilin-dependent γ-secretase complex at the so-called S3 site (reviewed in [9,10,11]). The promiscuous γ-secretase cleaves a large set of substrates, raising the question as to its regulation. It is known that membrane topology and composition influence Notch proteolysis, and hence Notch signaling activity. Substrate accessibility depends on the accurate distribution of substrate and protease in membrane microdomains, allowing for protease selectivity with regard to a given substrate, e.g., Notch (reviewed in [12,13,14]). Members of the highly conserved tetraspanin meshwork regulate the enzymatic activity of both proteases, notably of the ADAM family in mammals and *Drosophila* alike [15,16] (reviewed in [17]). Regulators of γ-secretase-mediated Notch cleavage identified in *Drosophila* are the transmembrane proteins Crumbs and Sanpodo. Crumbs is pivotal to apical-basal polarity in epithelia. By repressing γ-secretase activity, Crumbs induces a refinement of the Notch activity domain specifically along the presumptive wing margin [18]. In contrast, Sanpodo acts as a positive regulator of γ-secretase, and hence of Notch signaling, in the context of the asymmetric cell division of sensory organ precursors during the development of the peripheral nervous system [19,20] (reviewed in [21]).

As exemplified by the above examples, *Drosophila* has served as an exquisite model system for unraveling the Notch signaling pathway by genetic means. Mutations in many of the Notch pathway components display dominant phenotypes affecting, for example, the wing, the eye, or the mechano-sensory bristles, revealing a remarkable dose sensitivity [22]. This has allowed geneticists to screen for modifiers, and to uncover many regulators of the Notch signaling pathway (reviewed in [3,23]). For example, we used the overexpression of Hairless (H) in a systematic screen for Notch modifiers during eye development [24]. H has a major role in the silencing of Notch induced transcriptional activation, as it assembles repressor complexes together with Su(H) on Notch target genes (reviewed in [25,26]). Accordingly, overexpression of H results in extreme Notch loss of function phenotypes. Enhancers of this effect are expected to act similarly to Notch antagonists, whereas repressors should have a positive role in Notch signaling activity. During this screen, we identified EP555 as a repressor of the H-induced small eye phenotype. EP555 is an insertion line expected to drive expression of a nearby gene that should normally act as a Notch agonist. Here, we show that EP555 drives expression of the gene CG32521, encoding membrane-bound proteins that are broadly expressed during development. Based on its role to enhance Notch signaling activity, we renamed the gene to *mnr*, i.e., *membrane-bound Notch regulator*. We provide evidence that Mnr proteins support Notch signaling activity by easing the cleavage of the Notch receptor. This is, to our knowledge, the first general membrane-bound factor identified to directly augment Notch intramembrane processing, thereby acting as a positive regulator of Notch signaling activity.

## 2. Materials and Methods

### 2.1. Fly Work and Microscopy

For tissue-specific overexpression, the Gal4-UAS system was applied [27], using the driver lines gmr-Gal4 for expression in the developing eye [28], ptc-Gal4 for expression along the antero-posterior border [29], and Eq1-Gal4 (kind gift of H. Sun) for expression in the developing thorax [30]. Responder lines were UAS-*H* [31], UAS-*N* [32], UAS-*Su(H)* [33], UAS-*Dl^DN^* [34] and EP555 [35], and combinations thereof. Experiments on apoptosis used the lines gmr-*rpr* [36], gmr-*grim* [37], gmr-*hid* [38] (kind gifts of B. Hay) and gmr-*p53* [39], directly or combined with EP555. Flies were obtained from Bloomington Drosophila stock center BDSC if not noted otherwise. The fly eye-based reporter line for γ-secretase activity gmr-APP-Gal4 UAS-*grim*/CyO was a kind gift from M. Milán and B. Hay [18,40]. Either UAS-lacZ or UAS-GFP were used for controls. RNA interference was induced with lines v43699 and v43700 obtained from Vienna Drosophila Research Center VDRC [41]. Stocks were maintained at 18 °C on standard fly food; crosses were carried out at 25 °C. Analyses were on female flies if not noted otherwise.

Wings from adult flies were dehydrated in ethanol and mounted in Euparal (Roth, Karlsruhe, Germany). For microscopy, we used a Zeiss Axiophot (Zeiss, Jena, Germany). Fly heads and thoraces were visualized with a Wild M3Z stereo microscope (Leica, Wetzlar, Germany). Pictures were taken with a Pixera ES120 camera, using *Pixera viewfinder Pro2.5* software (Pixera, Santa Clara, CA, USA). Figures were assembled using Image J (open source), Corel photo paint and Corel draw software (Corel, Ottawa, ON, Canada). The *free hand tool* of Image J was used for size determination.

### 2.2. Analysis of CG32521 Expression

The cDNA clone RH54416 (NCBI accession number AY094937), encoding full-length RA transcript, was obtained from the Drosophila Genomics Resource Center DGRC (Bloomington, IN, USA). For in situ hybridization, it was labeled with DIG-dUTP by random priming using the DIG DNA Labeling and Detection kit (Roche, Merck, Darmstadt, Germany). Standard protocols were followed for in situ hybridization of whole mount embryos [42] and imaginal discs [43] with minor modifications in case of the latter. Notably, we omitted the deoxycholate step, and blocked proteinase K treatment by immersing tissues twice in 2 mg/mL glycine.

Polyclonal guinea pig anti-Mnr antibodies were raised against the full-length PA peptide (Genbank ID AAF50844, NCBI, Bethesda, MD, USA) fused to maltose binding protein MBP [44]. To this end, the coding sequence was PCR amplified, adding *Eco* RI and *Sal* I site, and inserted into pMal-c2 expression vector (NEB, Ipswich, MA, USA) in frame with N-terminal malE to generate pMal-Mnr. Primers used: UP555 EcoRI 5′ AGC GAA TTC ATG CTG GCA CTG CCT 3′; LP555 SalI 5′ GGA ATG CTA GTC GAC CTA CTT CAG TCG 3′. MBP-Mnr fusion protein was expressed in *E. coli*, purified on amylose resin (NEB, Ipswich, MA, USA) followed by maltose elution according to standard protocols, exactly as described before [44]. Immunization with purified MBP-Mnr fusion protein was performed by PINEDA ABservice (Berlin, Germany).

Antibody staining of S2 cells followed original protocols [45] with slight modifications. Cells were fixed for 15 min in 4% paraformaldehyde, washed with PBS and resuspended in PBTn (PBS plus 0.1% Tween 20 and 1% normal goat serum NGS). Incubation with antibodies was for 1 h at room temperature, followed by several washes in PBS. Primary antibodies used were guinea pig anti-Mnr (1:250), rabbit anti-GM130 (ab30637; Abcam, Cambridge, UK) (1:500), rabbit anti-Hook (1:500) (gift of H. Krämer) [46], as well as Rhodamin-coupled Phalloidin (R415, Thermo-Fisher Sci, Bonn, Germany) (1:200). Goat secondary antibodies, coupled to either FITC or Cy3, were from Jackson Laboratories (1:250) (Dianova, Hamburg, Germany).

Immunostaining of salivary glands were according to standard protocols. Glands were dissected from wandering third instar larvae in PBS, and fixed for 20 min with 4% paraformaldehyde. Washes were in PBT, pre-incubation steps and incubation included 4% NGS. Primary antibodies were added over night at 4 °C; secondaries (1:250) for several hours at room temperature in the dark. Primary antibodies were guinea pig anti-Mnr (1:500), mouse anti-arm (1:25) (N2.7A1; developed by E. Wieschaus, obtained from Developmental Studies Hybridoma Bank DSHB), Notch intracellular domain (C17.9C6) (1:25), and Delta extracellular domain (C594.9B) (1:25) (both developed by S. Artavanis-Tsakonas, from DSHB). Fluorescently labeled specimens were mounted in Vectashield (Vector labs, Eching, Germany) visualized using a BioRad confocal microscope MRC1024 with *LaserSharp 3.1* software (Bio-Rad, Hemel Hempstead, UK), supplemented with a Zeiss Axioskop (Zeiss, Jena, Germany).

To isolate proteins from fly heads, heads were separated from the fly body by shock-freezing in liquid nitrogen and hefty shaking. Protein extracts were prepared from 100 heads each in 100 µL binding buffer (20 mM HEPES pH 7.6, 150 mM KCl, 2.5 mM MgCl_2_, 10% glycerol, 0,05% Nonidet P-40, 1 mM DTT, plus one mini tablet cOmplete^TM^ ULTRA protease inhibitor cocktail (Roche Diagnostic; Rotkreuz, Switzerland) per 10 mL buffer), and analyzed via Western blot as outlined earlier [47,48]. The equivalent of about 5 heads was loaded per lane, and probed with rat anti-E-cadherin (1:100) (DCAD2, developed by T. Uemura, obtained from DSHB) and guinea pig anti-Mnr (1:500), with AP-coupled secondaries (1:1000) from Jackson Lab (Dianova, Hamburg, Germany). Five blots from two biological replicates were evaluated using *Image J gel* analysis tool (open source).

### 2.3. Protein Fractionation

Proteins were fractionated using the ProteoExtract^®^ subcellular proteome extraction kit (Merck-Millipore, Darmstadt, Germany) according to the manufacturer’s protocol. About 10^8^ cells were used for the assay. Fractionated proteins were PAGE-separated, blotted and probed with anti-Pzg (1:1000) [49], anti-Dlg (1:10) (4F3; developed by C. Goodman, obtained from DSHB), anti-beta tubulin (1:50) (E7; developed by M. Klymkowsky; obtained from DSHB) and guinea pig anti-Mnr antibodies (1:500), respectively.

To isolate membrane proteins, 30 mL of S2 cell culture were harvested by centrifugation at 200× *g* for 5 min at 4 °C. The pellet was washed twice in PBS, and once in 5 mL of MB buffer (400 mM sucrose, 40 mM Tris-HCl, 1 mM EGTA, 5 mM 2-mercaptoethanol, 10 mM KH_2_PO_4_, 0.2% BSA, pH 7.4), to be resuspended in MB buffer to a concentration of 5 × 10^7^ cells per 2 mL. Subsequent steps were on ice. The cells were allowed to swell for 20 min, and then homogenized in a tight Dounce glass homogenizer with 20 strokes. The lysate was centrifuged at 1400× *g* for 1 min at 4 °C. The pellet contained the nuclei. Mitochondria were removed with the pellet after centrifugation for 10 min at 4 °C and 8000× *g*. The supernatant was centrifuged for 20 min at 4 °C at 100,000× *g* twice. The pellets, enriched for membranes, were united, and stored at −20 °C. Peripheral proteins were removed by alkaline extraction in Na_2_CO_3_ (pH10) as described [50]. Integral membrane proteins were solubilized with Nonidet P-40 (Fluka AG, Buchs, Switzerland) (1% NP-40 in 50 mM Tris-HCl, pH 7.5), followed by a 20 min centrifugation step at 4 °C at 100,000× *g*. Samples from supernatants and pellets, respectively, were separated by PAGE, blotted and probed with anti-Mnr antibodies (1:500).

### 2.4. dsRNA Production against RA and RC

DNA specific for RA and RC was generated by PCR amplification of exons 2 and 5, respectively, with primers bearing a T7 promotor. The following primers were used: exon 2 UP 5′ TAA TAC GAC TCA CTA TAG GGA GCC ATA ACG CAT ACG CCC CGA 3′; exon 2 LP 5′ TAA TAC GAC TCA CTA TAG GGA AGA G AC CGC CAA GTA ATG TTA 3′; exon 5 UP 5′ TAA TAC GAC TCA CTA TAG GGA GAA CAA CAT CTT TGC ACC CGC 3′; exon 5 LP 5′ TAA TAC GAC TCA CTA TAG GGC GCG TTG GAG TGC TGG CTC TGA 3′. The obtained DNA was purified by agarose gel electrophoresis. RNA was produced with the RiboMax^TM^ T7 kit (Promega, Walldorf, Germany) according to the manufacturer’s protocol. The reaction was run for 4 h at 37 °C, and the DNA removed by DNase I treatment as suggested in the protocol. dsRNA was ethanol precipitated and stored at −20 °C. Concentration was determined by OD_260/280_ measurements. Integrity was confirmed by agarose gel electrophoresis.

### 2.5. Aggregation Assay of S2 cells

Maintenance of S2 cells, aggregation assays and antibody staining were performed as outlined before [45,51]. The cell lines S2 (CVCL_TZ72), S2-Mt-N (CVCL-0A47), and DeltaWTNdeMYC (CVCL-0Q78) were obtained from the Drosophila Genomics Resource Center DGRC. The latter are stably transfected with full-length pMt-Notch and pMt-Delta-MYC, respectively, expression of which was induced by overnight incubation with 0.7 mM CuSO_4_. Aggregate formation was allowed in a 2 mL volume in a 6-well cell-culture plate for 30 min on a belly dancer. 60 µL of cells were transferred to poly L-Lysine treated slides (Thermo-Fisher Sci, Bonn) into a frame-seal chamber, and incubated for further 15 min before fixation with 2% paraformaldehyde followed by light microscopy or additional antibody staining. For antibody staining, mouse monoclonals directed against Notch intracellular domain (1:25) (C17.9C6) (developed by S. Artavanis-Tsakonas, obtained from DSHB) and rabbit anti-MYC (1:50) (Sigma Aldrich, Merck, Darmstadt, Germany) were used. Secondary antibodies coupled to FITC or Cy3 (1:250) were obtained from Jackson lab. RNAi was induced by incubating cells with 10^6^ cells/ml density with respective dsRNA at 37 nM final concentration as outlined before [52]. Aggregation assays were performed three days after RNAi treatment.

### 2.6. Notch Activity Assay in S2 Cells

Notch activity was determined with a luciferase assay as described before [33,53], using the Promega Dual-luciferase^®^ reporter assay system (Promega, Walldorf, Germany). The Notch response element NRE, driving firefly luciferase expression, served as reporter; activity was normalized to Renilla luciferase. Cells at 2.5 × 10^6^ density were transfected using SuperFect^®^ transfection reagent according to the manufacturer’s protocol (Qiagen, Hilden, Germany). Plasmids were added as follows NRE (1 µg), pRL-TK (0.2 µg), pMT-Notch (1 µg) [45], pMT-Mnr (0.5 µg; shuttled from pMal-Mnr into pMT-vector with *Eco* RI/*Sal* I) plus empty pMT plasmid [54] up to 5 µg. Subsequently, dsRNA (15 µg each of exon 2 and exon5) was added, and cells rested for three days to allow RNAi to occur. Subsequently, Notch and Mnr expression was induced by CuSO_4_ treatment overnight, followed by several washes and a 30 min EDTA treatment (2 mM in PBS) for Notch receptor activation. Cells were then washed for a complete removal of EDTA before measuring Luciferase activity.

Efficacy of RNAi mediated knockdown of *mnr* expression in S2 cells was determined by qRT-PCR as outlined earlier [55]: poly(A)^+^ RNA was isolated with the Dynabeads^TM^ *mRNA DIRECT^TM^ Purification Kit* (Invitrogen, Thermo Fisher Scientific, Waltham, MA, USA) from 3 mL of S2 cells subjected to RNAi for three days as outlined above; untreated cells served as controls. mRNA was digested with DNAse I (New England Biolabs, Frankfurt, Germany) and reverse transcribed with *qScriber cDNA Synthesis Kit* (highQu, Kraichtal, Germany). Real time qPCR was conducted with *Blue S’Green qPCR kit* (Biozym, Hessisch-Oldendorf, Germany) using MIC magnetic induction cycler (*bms*, Pots Point, Australia) including target and no-template controls. Absence of genomic DNA was confirmed in a non-RT control. As internal references for *mnr* expression, *cyp33* and *Tbp* were selected based on variance and Cq values. Relative quantification of the data, obtained from three biological and two technical replicates, was with *micPCR* software Version 2.10.5 based on *REST* taking target efficiency into account [56]. The *mnr* primer pair *mnr_Exon7_1* spans exon 7, and hence detects all transcripts (5′ - > 3′): TTT GAT CCA AGG GCG TGA CA and AGT AGG CGT CCA CGG AGT AG. Primer pairs for the references were *cyp33* PP14577 and *Tbp* PP1556 from DRSC FlyPrimer bank [57].

### 2.7. Bioinformatics and Statistics

Information on the CG32521 locus was fetched from flybase (FB2021_04, released 17 August 2021). For in silico analysis of Mnr protein, analysis tool web services from the EMBL-EBI were used (https://www.ebi.ac.uk/Tools/webservices/, accessed on 25 June 2021) [58], applying *EMBOSS Pepstats* for amino acid distribution, pI and molecular weight, *EMBOSS Pepinfo* for hydropathy Plots using to Kyte & Doolittle parameters and *Phobius* programs. O-glycosylation was predicted with *NetOGlyc-4.0* (https://services.healthtech.dtu.dk/service.php?NetOGlyc-4.0, accessed on 10 June 2021) [59]. Box plots were generated with *BoxPlotR* (http://shiny.chemgrid.org/boxplotr/, accessed on 22 October 2021). Data were sampled and exploited with MS Excel. Quantifications were rated for normality using the Anderson-Darling normality test (http://in-silico.online/, accessed on 29 September 2021). Statistical significance was assessed with ANOVA for multiple comparisons using a two-tailed Dunnett’s approach in case of a comparison of several samples with a single control, and using a two-tailed Tukey–Kramer approach for comparisons of every sample with every other sample; *** *p* < 0.001, ** *p* < 0.01, * *p* < 0.05, not significant ns *p* > 0.05.

## 3. Results and Discussion

### 3.1. EP555 Drives Expression of CG32521 Supporting Notch Signaling Activity with an Apparent Anti-Apoptotic Role in Eye Development

**Figure 1:**
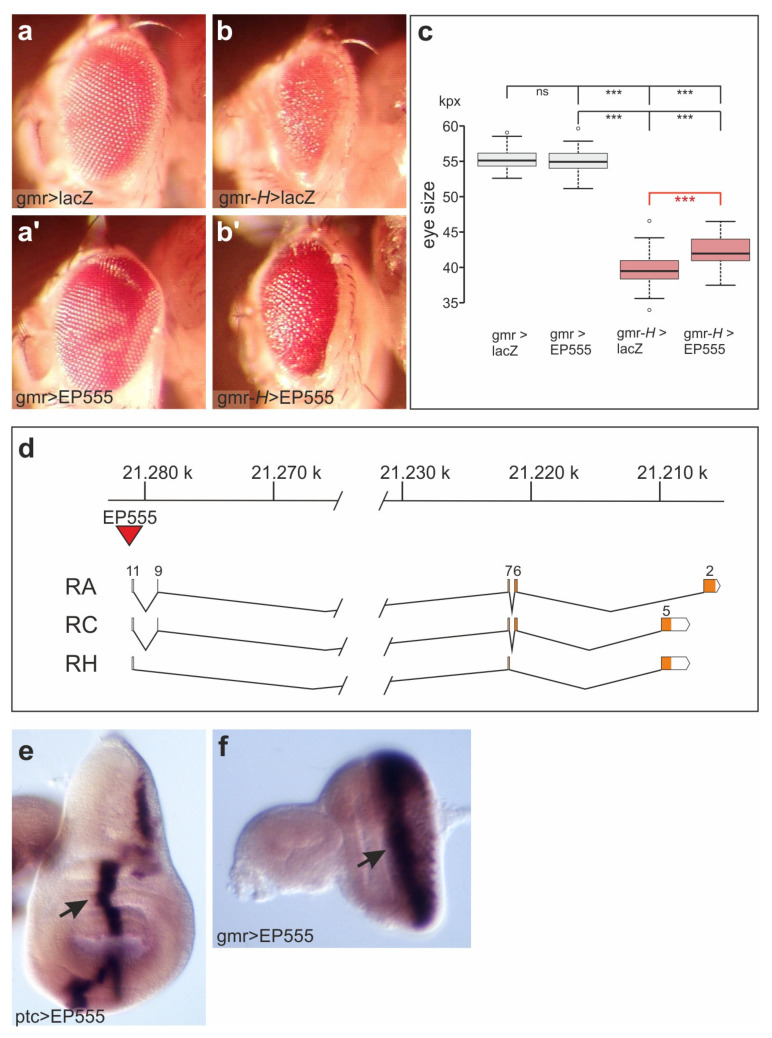
EP555 rescues gmr>*H* and drives expression of CG32521. (**a**–**b’**) Overexpression of the indicated genes during eye development induced with gmr-Gal4. Note increased eye size when combining UAS-*H* with EP555. Genotypes: gmr-Gal4/+; UAS-lacZ/+ (**a**); EP555/+; gmr-Gal4/+ (**a’**), gmr-Gal4 UAS-*H*/+; UAS-lacZ/+ (**b**), EP555/+; gmr-Gal4 UAS-*H*/+ (**b’**). (**c**) Quantification of the EP555 rescue effect; female eye size was determined (*n* = 30, relevant data are shown in red). Values are presented as boxplots; center lines show the medians, box limits indicate the 25th and 75th percentiles (R-software, version 0.5.0, 2013); whiskers extend 1.5 times the interquartile range; outliers are represented by dots. Statistical significance was determined by ANOVA two-tailed Tukey–Kramer’s approach (*** *p* < 0.001, not significant ns *p* > 0.05). The rescue effect of EP555 on gmr-*H* induced eye size is highly significant with a *p*-value of 0.0003 as determined by Student’s t-test. (**d**) Scheme of the CG32521 locus, scale is in kb. The locus extends over 70 kb, encoding nine transcripts. Three transcripts (RA, RC and RH) representing the two major classes are shown; exons are numbered according to flybase (FB2021_04, released 17 August 2021). The coding region is highlighted in orange. The position of the EP555 insertion is indicated (red triangle). (**e**) Expression of CG32521 along the antero-posterior border, detected by in situ hybridization in wing imaginal discs driving EP555 with ptc-Gal4 (arrow). (**f**) Likewise, CG32521 expression detected in eye-antennal discs driving EP555 with gmr-Gal4 (arrow).

In a screen for genetic modifiers of the Notch antagonist Hairless (H), we identified EP555 as a potential suppressor of H activity [24]. Whereas overexpression of EP555 alone was without phenotypic consequences, the small and rough eye phenotype following overexpression of H was significantly ameliorated (Figure 1a–c). EP555 is inserted 43 bp upstream of the CG32521 transcription start site (Genbank ID AQ025349), localized at the base of the X-chromosome in 19F3. Accordingly, tissue specific overexpression of EP555 induced the transcription of CG32521, demonstrating that this EP-line indeed drives CG32521 expression (Figure 1d–f). The function of H is to antagonize Notch signaling activity [25]. Hence, we wondered whether CG32521 may be involved in the regulation of the Notch pathway.

**Figure 2:**
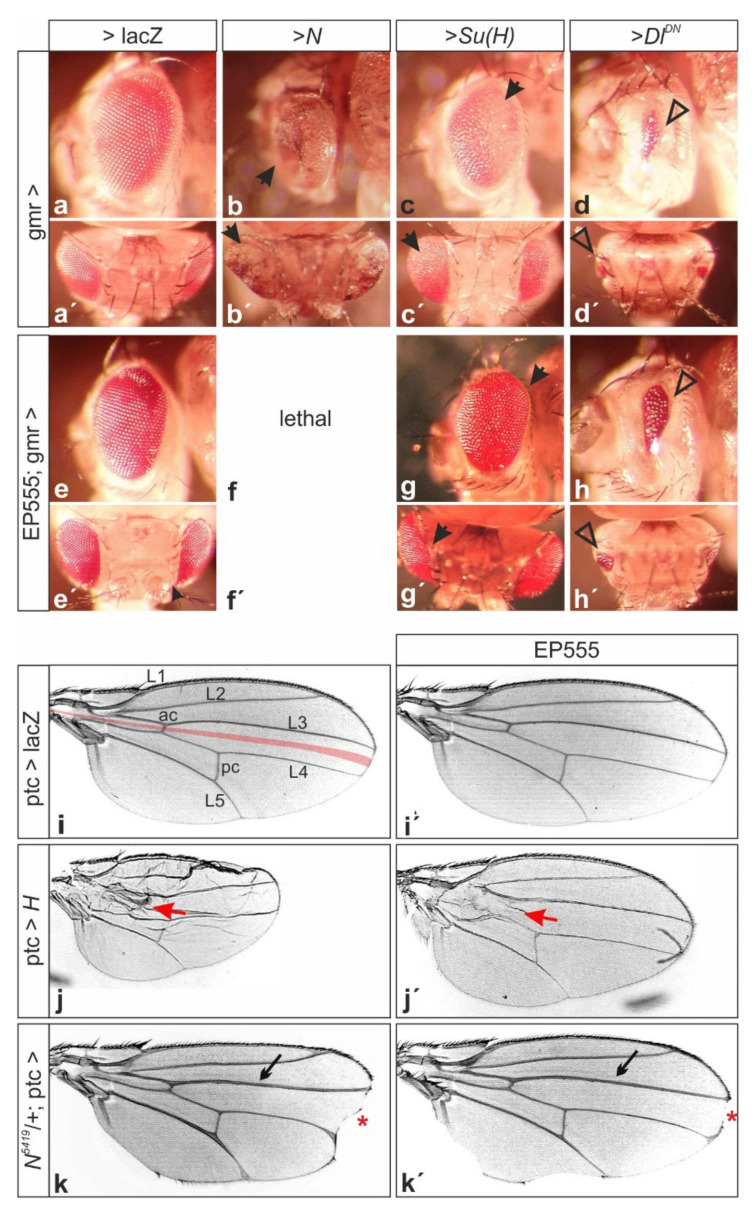
EP555 acts as a modifier of Notch pathway components. Lateral view (**a**–**h**), and top view (**a’**–**h’**) of female adult heads of the indicated genotypes. (**a**–**d’**) Overexpression with gmr-Gal4 results in an overgrowth of the eye driving *Notch* (**b**,**b’**) or *Su*(*H*) (**c**,**c’**) (arrows), and reduces eye size driving dominant negative *Delta* (**d**,**d’**) (arrowheads). (**e**–**h’**) Co-overexpression of EP555. Note lethality in combination with *Notch* (**f**,**f’**), enhancement of *Su(H)* gain of function phenotype (arrows in **g**,**g’**), and rescue of dominant negative *Delta* (arrowheads in **h**,**h’**). (**i**–**k’**) Representative wings from female flies are shown. (**i**) Control wing; ptc-Gal4 drives expression along the antero-posterior boundary in the wing blade, indicated in red. Longitudinal veins L1-L5, anterior cross vein (ac) and posterior cross vein (pc) are labeled. (**i’**) EP555 expression is without apparent phenotype. (**j**) Overexpression of *H* results in smaller wings; note defects in the anterior cross vein (red arrow). (**k**) Heterozygous *N^5419^* mutants display typical wing notches (red asterisk) and thickened veins (small arrow). (**j’**–**k’**) Combined with EP555 the respective phenotypes are ameliorated.

To address a possible influence of CG32521 on the regulation of Notch signaling activity, EP555 was overexpressed in combination with Notch pathway components. In agreement with a positive role of CG32521 in the Notch signaling pathway, the overproliferation induced by Su(H) overexpression in the developing eye was enhanced by EP555; a combination with activated Notch even caused pupal lethality. Moreover, the small eye phenotype elicited by the overexpression of a dominant negative version of the Notch ligand Delta was rescued as expected (Figure 2a–h’). The genetic interactions between EP555 and Notch pathway members were not restricted to eye development as they were also observed in the wing. For example, a local overexpression of EP555 along the antero-posterior boundary within the developing wing rescued the effects of a likewise overexpression of the Notch antagonist H as well as of a loss of Notch activity (Figure 2i–k’).

**Figure 3:**
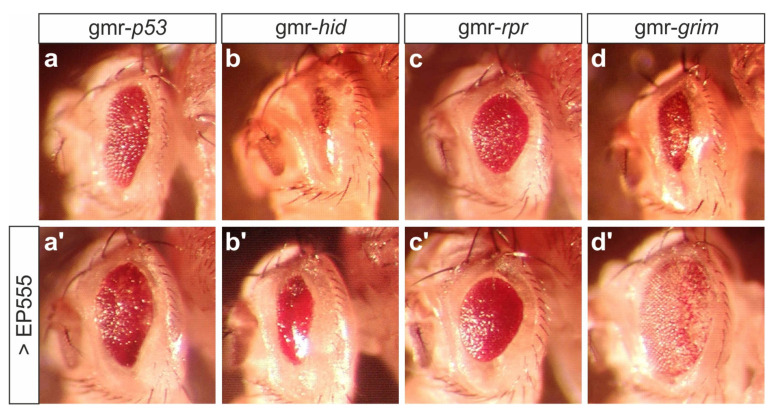
EP555 attenuates pro-apoptotic gene activity. (**a**–**d’**) Overexpression of the indicated genes during eye development, induced with gmr-Gal4. Female heads are shown. Note increased eye size in combination with EP555. Genotypes: gmr-*p53*/+ (**a**), EP555/gmr-*p53*; gmr-Gal4/+ (**a’**), gmr-*hid*/+ (**b**), EP555/+; gmr-*hid*/gmr-Gal4 (**b’**), gmr-*rpr*/+ (**c**), EP555/+; gmr-Gal4/+; gmr-*rpr*/+ (**c’**), gmr-*grim*/+ (**d**), EP555/+; gmr-Gal4/+; gmr-*grim*/+ (**d’**).

We have shown before that the small eye phenotype induced by the overexpression of H results from apoptosis during eye development [24,60]. Accordingly, the eye defects resulting from an induction of the pro-apoptotic genes *p53*, *hid*, *rpr* and *grim* were considerably ameliorated by the concurrent EP555 overexpression (Figure 3). Notch signaling activity is instrumental to the control of growth and cell death [2,61,62,63,64]. Accordingly, a Notch agonist may be expected to counteract pro-apoptotic gene activity, for example by promoting cell proliferation. Together, we conclude that CG32521 acts as a positive factor in the Notch signaling pathway with an anti-apoptotic role during *Drosophila* eye development.

### 3.2. CG32521 Provides Several Transcript Classes with a Broad Expression during Embryogenesis

A total of nine different transcripts derive from CG32521 (flybase FB2021_04, released 17 August 2021). Differential splicing results in two major classes of transcripts that primarily differ in their last exons. Prime examples are shown in Figure 1d. Whereas transcript class RA ends with exon 2, differences are observed in the use of non-coding exons. RC and RH both end with exon 5 instead of exon 2, but RH lacks the coding exon 6, which is common to class RA and RC. All transcripts share exon 7 encoding the N-terminal translation start (Figure 1d). Exon numbering is according to flybase. CG32521 is only conserved in insects (flybase FB2021_04, released 17 August 2021).

**Figure 4:**
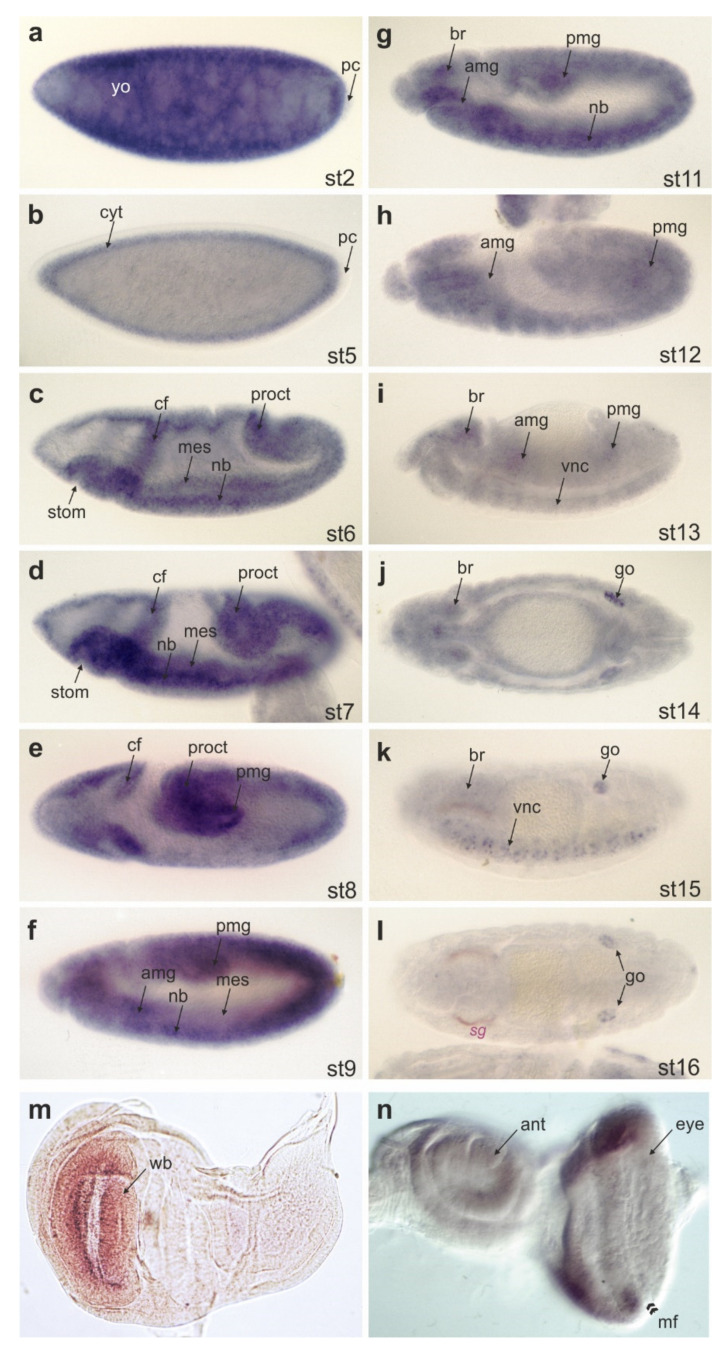
Expression of CG32521 during development. In situ hybridization to detect CG32521 transcription during development. (**a**–**l**) Embryos are oriented anterior to the left and dorsal up except for the top view in (**j**) and (**l**); developmental stages (st) are indicated. Note enrichment in the yolk (yo) (**a**), the mesoderm (mes) (**c**–**f**), the developing central nervous system and the brain (nb, br) (**c**–**k**), as well as the gonads (go) (**j**–**l**). In the wing imaginal disc, transcripts accumulate in the presumptive wing blade (wb); arrow in (**m**), whereas in the eye-antennal disc, an accumulation is observed in the proliferating part anterior to the morphogenetic furrow (mf); arrowheads in (**n**). Abbreviations are: amg, anterior midgut; ant, antennal disc; br, brain; cf, cephalic furrow; cyt, cytoplasm; eye, eye disc; go, gonads; mes, mesoderm; mf, morphogenetic furrow; nb, neuroblasts; pc, pole cells; pmg, posterior midgut; proct, proctodaeum; sg, salivary glands; stom, stomodaeum; vnc, ventral nerve chord; wb, wing blade; yo, yolk.

During early embryogenesis, CG32521 is expressed in all germ layers as revealed by in situ hybridization of cDNA RH54416, corresponding to RA but detecting all transcripts with exon 6, i.e., seven out of nine (Figure 4). Midway through embryogenesis from stage 12 on, expression ceases, and transcripts accumulate at low levels primarily within the developing central nervous system and the gonadal mesoderm. In wing imaginal discs, CG32521 transcripts are enriched in the presumptive wing blade. Expression in eye-antennal discs appears largely restricted to the three presumptive antennal segments and the anterior, proliferating part of the eye anlagen (Figure 4).

### 3.3. CG32521 Encodes Membrane-Bound Proteins

**Figure 5:**
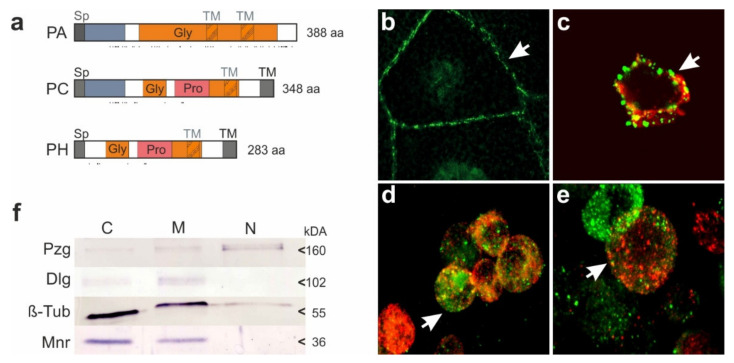
CG32521 encodes membrane-bound proteins Mnr. (**a**) Sketch of presumptive proteins PA, PC and PH encoded by CG32521. Size is to scale, amino acids (aa) are given. All proteins contain a signal peptide (Sp), and Glycine rich domains (Gly). Moreover, PA and PC share the N-terminal 88 amino acids (blue). In addition, PC and PH are enriched in Proline residues (Pro). Predicted transmembrane domains (TM) are shaded in grey. Potential O-glycosylation sites are marked below by dashes. PA has a predicted molecular weight of 36.5 kDa; PC of 35.06 kDA and PH of 28.5 kDa. (**b**–**e**) Antibodies directed against Mnr (green) outline the plasma membrane of salivary gland cells (arrow) (**b**). In Schneider S2 cells, membranes are stained as well within dotted structures that co-localize with Phalloidin (**c**), and with the Golgi markers Hook (**d**) and GM130 (**e**). (**f**) Cytoplasmic C, membrane M, and nuclear N fractions of S2 cells reveal accumulation of Mnr in the cytosol and membrane. As reference, the nuclear protein Pzg, the transmembrane protein Dlg and beta-tubulin were detected. Approximate protein size is given in kDa.

The proteins encoded by CG32521 are rather small and neutral, with a predicted molecular weight of around 30 kDA (Figure 5a). All contain a signal peptide at the shared N-terminus, encoded by exon 7 (Figure 1a). A trans-membrane domain; however, is robustly predicted for PC and PH at the very C-terminus. Accordingly, this protein class is expected to extend extracellularly. A presumptive second trans-membrane domain close by suggests a trans-membrane loop directed towards the cytosol. Trans-membrane predictions in PA have a lower score; depending on the program used, one or two transmembrane domains are predicted (Figure 5a, Supplementary Figure S1). The encoded CG32521 proteins are highly enriched in Glycine residues (23–32%) and Proline residues (9–12%) clustered in the center, with about twofold the normal frequency. Moreover, numerous Tyrosine residues are found scattered all over that appear with about 1.5-fold the normal frequency. Notably PA protein may be highly O-glycosylated, common to membrane glycoproteins (Figure 5a). 

In agreement with the in silico predictions, both protein classes encoded by CG32521, proteins PA and PH, were identified in a membrane-enriched protein fraction by mass spectrometry from fly heads [65]. Polyclonal antibodies generated against PA and PC detected proteins enriched along the cell membranes in salivary glands of third instar larvae (Figure 5b). Moreover, S2 cells revealed dotted staining along the cell’s outline and some overlap with Golgi-markers in agreement with a trans-membrane localization (Figure 5c–e). Fractionation of S2 cells confirmed an enrichment in the membrane fraction (Figure 5f and Supplementary Figure S1). Based on the above findings, we renamed CG32521 to *mnr* for *membrane-bound Notch regulator*, encoding Mnr proteins.

### 3.4. The Membrane-Bound Notch Regulator Mnr Encoded by CG32521 Boosts Notch Signaling Activity

**Figure 6:**
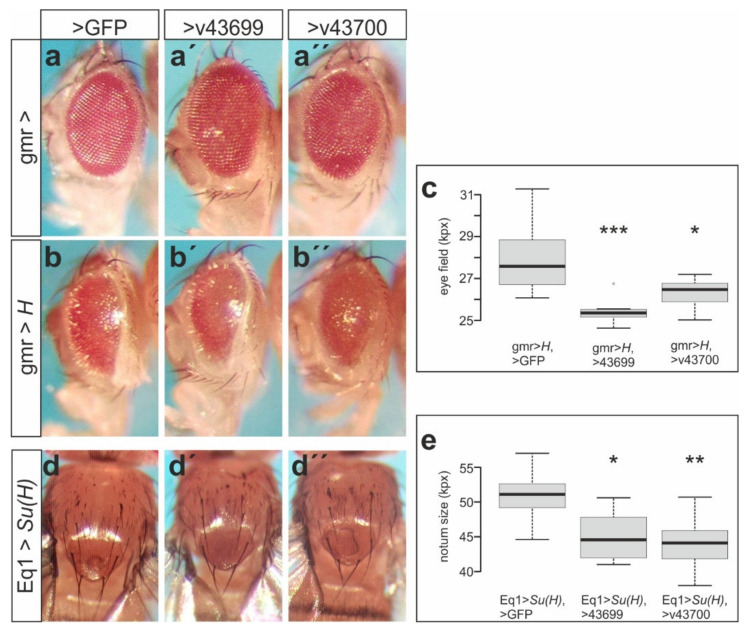
Mnr regulates Notch activity. Knockdown of *mnr* activity by RNA interference influences Notch signaling output. Female flies are shown. (**a**–**a’’**) Overexpressed in the developing eye, two independent RNAi lines (v43699, v43700) have little impact on eye development. (**b**–**b’’**) Both lines significantly improve the small eye phenotype resulting from H overexpression. (**c**) Statistical evaluation of eye size from females of the given genotype (*n* = 8). Boxplots with center lines indicating the medians and box limits the 25th and 75th percentiles (R-software); whiskers extend 1.5 time the interquartile range; outliers are represented by dots. (**d**–**d’’**) Size increase following Su(H) overexpression in the center of the presumptive thorax (**d**) is reduced by concurrent downregulation of *mnr* (**d’**,**d’’**). (**e**) Statistical evaluation of thorax size from females of the given genotype. The area between the dorso-central bristles up to the prothorax was measured. Boxplots are as in (**c**); *n* = 8. (**c**,**e**) Significance was tested by ANOVA two-tailed Dunnett’s approach for multiple comparisons relative to UAS-GFP-control (*** *p* < 0.001, ** *p* < 0.01, * *p* < 0.05). Genotypes: gmr-Gal4/+; UAS-GFP/+ (**a**), gmr-Gal4/+; UAS-v43699/+ (**a’**), gmr-Gal4/+; UAS-v43700/+ (**a’’**), gmr-Gal4 UAS-*H*/+; UAS-GFP/+ (**b**), gmr-Gal4 UAS-*H*/+; UAS-v43699/+ (**b’**), gmr-Gal4 UAS-*H*/+; UAS-v43700/+ (**b’’**), Eq1-Gal4 UAS-*Su(H)*/+; UAS-GFP/+ (**d**), Eq1-Gal4 UAS-*Su(H)*/+; UAS-v43699/+ (**d’**), Eq1-Gal4 UAS-*Su(H)*/+; UAS-v43700/+ (**d’’**).

Our results so far suggest that the Mnr proteins act as positive regulators in the Notch signaling pathway. In this case, we expected a downregulation of CG32521 to impede Notch activity as well as to enhance the activity of the Notch antagonist H. Accordingly, a small deficiency Df(1)Q359 overlapping the *mnr* locus enhanced the gmr-*H* phenotype as expected [24]. This deficiency, however, also deletes many other genes. Taking into consideration the exquisite sensitivity of the Notch pathway to genetic background, we decided to apply RNA interference in order to knockdown *mnr* activity specifically. We made use of two RNAi lines from VDRC, v43699 and v43700, that are directed against the last exon, i.e., transcript class RA only [41]. Although expression of RA was just partly knocked down to about 60% of control, a slight roughening of the eye was observed by the overexpression of these RNAi lines in the differentiating eye field with gmr-Gal4 (Figure 6a–a’’, Supplementary Figure S2). As expected, the small eye phenotype induced by gmr-*H* was enhanced by the knockdown of *mnr* (Figure 6b,c) in support of our hypothesis of a positive role of *mnr* in Notch signal transduction. In agreement, the overproliferation of the notum induced by Su(H) overexpression with Eq1-Gal4 [66] was ameliorated by a knockdown of *mnr* (Figure 6d,e).

**Figure 7:**
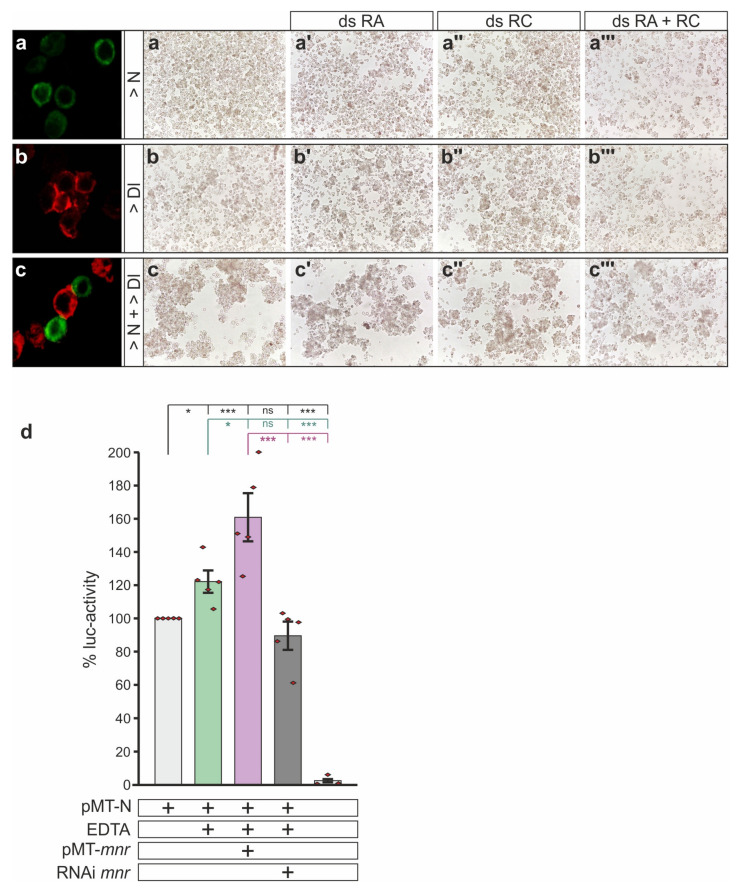
Mnr stimulates Notch receptor activation. (**a**–**c’’’**) Aggregation assay using S2 cells carrying either construct encoding full-length Notch (**a**), Delta (**b**), or both (**c**), plus double-stranded RNA of RA (**a’**,**b’,c’**), of RC (**a’’**,**b’’**,**c’’**), or both (**a’’’**,**b’’’**,**c’’’**). The left panel shows confocal antibody staining against Notch (green) and Delta (red) to demonstrate membrane localization and homo- and heterotypic aggregation of the cells. The other pictures are bright field. There is no apparent influence of a knockdown of *mnr* activity on the aggregation of S2 cells. (**d**) Luciferase assay as a read out of Notch receptor activation. S2 cells were transfected with full-length Notch, and the receptor was activated by EDTA treatment as indicated, resulting in NRE-reporter activation. Co-transfection with *mnr* cDNA increased, and knockdown of *mnr* activity by RNAi against RA and RC decreased Notch signaling output. NRE activation is reported by luciferase (luc) activity, given in comparison to Notch-transfected cells without EDTA activation. Values are presented as mean ± SD from five experiments. Significance was assessed with ANOVA two-tailed Tukey–Kramer test for multiple comparisons; depicted relative to pMT-N (upper row), relative to pMT-N activated with EDTA (middle row, green) and relative to pMT-N activated with EDTA plus pMT-Mnr (lower row, lilac), as indicated (*** *p* < 0.001, * *p* < 0.05, ns not significant *p* > 0.05).

Mnr proteins are membrane-bound mostly directing outside the cell. Based on this localization, Mnr proteins may support Notch signaling activity by a direct interaction with either the Notch receptor or the Delta ligand at the membrane, perhaps stabilizing their interaction. Accordingly, a co-localization of the Mnr, Dl and Notch proteins was seen in situ at the cell membranes of salivary glands, albeit not well resolved at this magnification (Supplementary Figure S3). To address the question, whether Mnr may ease the binding of Notch and Delta, we applied an aggregation assay using S2 cells. S2 cells normally grow in suspension [67], and do so as well, when transfected with full-length Notch constructs [45] (Figure 7a). In contrast, some homotypic adherence can be observed when S2 cells express Delta at their surface [45] (Figure 7b). Mixing of Notch- and Delta-expressing cells causes heterotypic attachment and formation of large cell clusters based on receptor–ligand interaction [45] (Figure 7c). The assay was performed while knocking down either RA and/or RC transcripts. The combined knockdown of RA and RC in S2 cells resulted in a more than 85% downregulation of *mnr* expression levels compared to untreated cells (Supplementary Figure S4). However, no influence on homo- or heterotypic cell clustering was observed (Figure 7a–c’’’), indicating that Mnr proteins have little impact on receptor–ligand interaction. Next, we assayed Notch signaling activity directly using a luciferase-based reporter assay. In this assay, luciferase expression is driven by the Notch Response Element NRE containing Su(H) binding sites, i.e., luciferase activity directly reports Notch activity [53]. This assay can be used for example to measure the influence of activator or repressor molecules on Notch mediated expression [33,53,68]. S2 cells were transfected with full-length Notch, and the receptor was subsequently activated by EDTA treatment [69]. This made it possible to directly assay the influence of Mnr on Notch receptor activation, rather than the transcriptional regulation. Indeed, a concurrent induction of *mnr* cDNA RA resulted in a significant increase in luciferase activity, whereas RNAi mediated downregulation of *mnr* resulted in a strong decrease (Figure 7d). These results show unequivocally that Mnr acts within the Notch signal-receiving cell, where it has a positive role in Notch signal transduction, perhaps by fostering Notch receptor cleavage and activation.

### 3.5. Mnr Promotes Notch Receptor Cleavage

**Figure 8:**
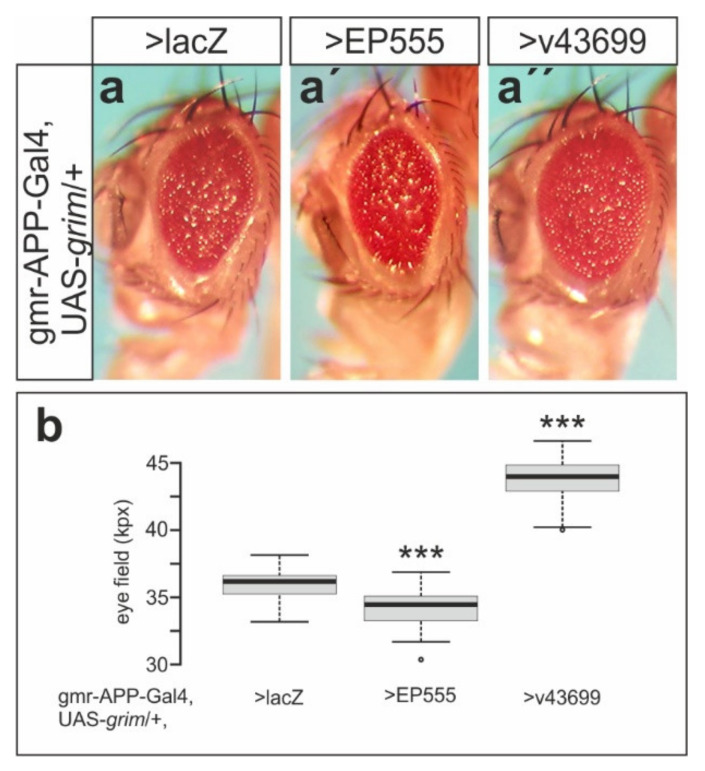
Mnr promotes γ-secretase activity. Activity of γ-secretase was monitored using a fly eye-based reporter system. Cleavage results in Gal4 release and expression of *grim*, inducing apoptosis in the developing eye. (**a**) Cell death observed in the UAS-lacZ control reveals endogenous γ-secretase activity. (**a’**) Concurrent expression of EP555 results in phenotype enhancement, (**a’’**) whereas *mnr* knockdown, induced by v43699 expression, ameliorates it. (**b**) Statistical evaluation of *mnr* influence on γ-secretase activity. The size of the female fly eyes was determined, and is represented by boxplots, with center lines depicting the medians and box limits indicating the 25th and 75th percentiles, as determined by R software; whiskers extend 1.5 times the interquartile range from the 25th and 75th percentiles, and outliers are represented by dots (*n* = 46, 43, 40). Significance was tested by ANOVA two-tailed Dunnett’s approach for multiple comparisons relative to UAS-lacZ-control (*** *p* < 0.001).

The outside directed trans-membrane position of Mnr proteins, and their role in supporting Notch receptor activation within the signal-receiving cell, suggested to us Mnr may regulate the cleavage of the Notch receptor. To test this possibility experimentally, we made use of an in vivo γ-secretase activity assay developed earlier [40]. This assay is based on a γ-secretase reporter construct APP-Gal4 expressed in the developing eye, where Gal4 is appended internally to the C-terminus of APP. Because the APP peptide is C-terminal to the S2 cleavage site, the yeast transcription factor Gal4 is released only upon γ-cleavage at the S3 site [40]. The APP-Gal4 strain is combined with pro-apoptotic UAS-*grim*, which is under transcriptional control of Gal4. Accordingly, small eyes result from apoptosis upon cleavage of APP-Gal4 by γ-secretase. The eye size of these reporter flies correlates directly with the level of endogenous γ-secretase activity, responding to changes of regulators in a highly sensitive manner [40]. In this genetic background, we induced an upregulation of *mnr* by introducing EP555, and a downregulation by adding the RNAi line v43699, and monitored eye size. Indeed, we observed significantly smaller eyes upon the upregulation of *mnr*, and a remarkable rescue of eye size upon its downregulation (Figure 8). Obviously, γ-secretase activity responds to Mnr levels: high levels result in an increase and low levels in a decrease. Apparently, γ-secretase function requires Mnr since cleavage is suppressed by the knockdown of *mnr* (Figure 8). How could Mnr promote γ-secretase activity? Mnr may stabilize the docking of Notch, or it may support the conformational changes of γ-secretase preceding proteolysis (reviewed in [9,12,14]). Perhaps, Mnr influences membrane topology, or the relative subcellular compartmentalization of γ-secretase components and Notch, known to be linked to substrate specificity (reviewed in [12,13,14,17]). Alternatively, Mnr may bias the cleavage site preference of γ-secretase. Normally, heterogeneous NICD fragments with varying N-termini arise that differ in their half-life [70]. Assuming Mnr facilitates cleavage towards the stable variant at the expense of the more labile versions, Notch signaling activity is expected to increase.

Another example for a γ-secretase regulator in *Drosophila* is *sanpodo*, encoding a four-pass transmembrane protein that promotes Notch signaling activity by interacting with the γ-secretase complex. Genetic analyses support a role in intramembrane cleavage of Notch, albeit direct experimental evidence is lacking [19,20]. *sanpodo* is required exclusively during the asymmetric cell division [21], whereas Mnr is not cell-type specific. Neither is conserved outside of insects, but similar mechanisms controlling γ-secretase activity, perhaps more complex than in insects, are expected in vertebrates as well.

## 4. Conclusions

**Figure 9:**
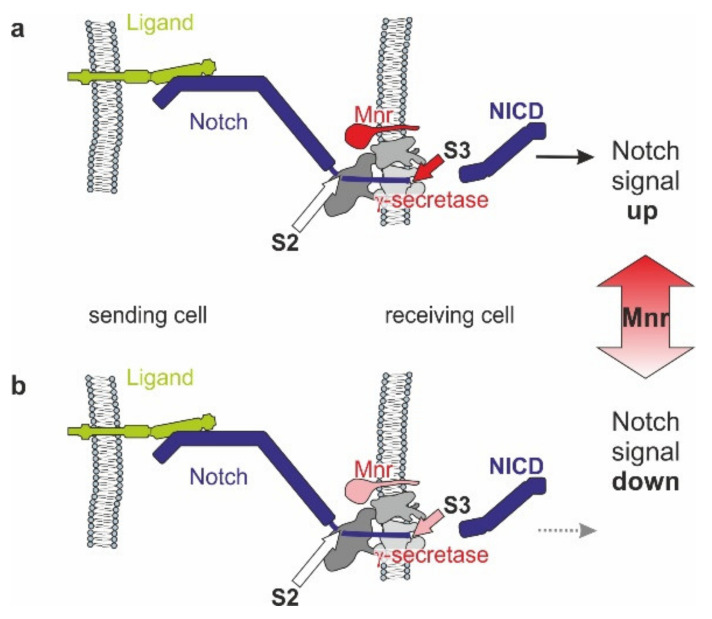
Role of Mnr in Notch signaling. (**a**) The sending cell (left) presents the Notch ligand, which binds to the Notch receptor on the signal-receiving cell. The resultant pulling force opens up the S2 cleavage site within the Notch-repressor region for the ADAM metalloprotease. In consequence of extracellular proteolysis, γ-secretase (in grey, composed of four subunits [71]) induces S3 cleavage of the Notch receptor within the membrane, thereby releasing the biologically active NICD fragment conveying the Notch signal. Mnr (in red) supports γ-secretase activity. (**b**) In case of decreased Mnr activity, γ-secretase activity is lowered and so is Notch signal transduction. Hence, increased Mnr levels cause Notch signals to go up, whereas lower Mnr levels reduce Notch signaling activity.

Notch signal transduction requires intra-membrane proteolysis of the Notch receptor. It is well appreciated that modifications of either ligands or the Notch receptor itself greatly influence their binding, and are accordingly an important mechanism in the regulation of Notch signaling activity (reviewed in [9,10]). Regulators of intramembrane cleavage; however, remain largely enigmatic [14]. With Mnr, we have identified an amplifier of Notch signal transduction. Mnr encodes a membrane-bound factor augmenting Notch intra-membrane processing, thereby acting as a positive regulator of Notch signaling activity (Figure 9). Although not conserved in vertebrates, similar mechanisms controlling γ-secretase activity are to be expected there as well. Based on its trans-membrane localization extending mostly outside the cell, Mnr proteins may be involved, for example, in the stabilization of Notch docking at the γ-secretase complex, in the subcellular compartmentalization of γ-secretase components or in biasing γ-secretase site preference [9,10,12,13,17,70]. Altogether, we speculate that the role of Mnr is to assist the intramembrane cleavage of the Notch receptor upon ligand binding, eventually promoting the Notch response (Figure 9).

## Data Availability

The data presented in this study are available in the article and the Supplementary Materials.

## Supplementary Materials

The following are available online at https://www.mdpi.com/article/10.3390/biom11111672/s1, Figure S1: Mnr encodes two classes of membrane-bound proteins, Figure S2: RNAi mediated knockdown of mnr expression by v43699 overexpression in the eye, Figure S3: Co-localization of Mnr protein with Notch and Delta, Figure S4: RNAi mediated knockdown of mnr expression in S2 cells.
