# Supplementary material for: The Membrane-Bound Notch Regulator Mnr Supports Notch Cleavage and Signaling Activity in Drosophila melanogaster
Source: Biomolecules. 2021 Nov 10;11(11):1672. doi: 10.3390/biom11111672 (PMC8615698; doi:10.3390/biom11111672)
Supplement: Supplementary file 1 [file biomolecules-11-01672-s001.zip › biomolecules-1396142-supplementary.pdf]

biomolecules

MDPI

Supplementary Materials to

The Membrane-Bound Notch Regulator Mnr Supports Notch Cleavage and Signaling Activity in *Drosophila melanogaster*

Anja C. Nagel *, Dominik Müller, Mirjam Zimmermann and Anette Preiss

Department of General Genetics 190g, University of Hohenheim, Garbenstr. 30, 70599 Stuttgart, Germany; muelled@gmx.de (D.M.); zimmermann.mirjam@uni-hohenheim.de (M.Z.); a.preiss@uni-hohenheim.de (A.P.)
* Correspondence: anja.nagel@uni-hohenheim.de

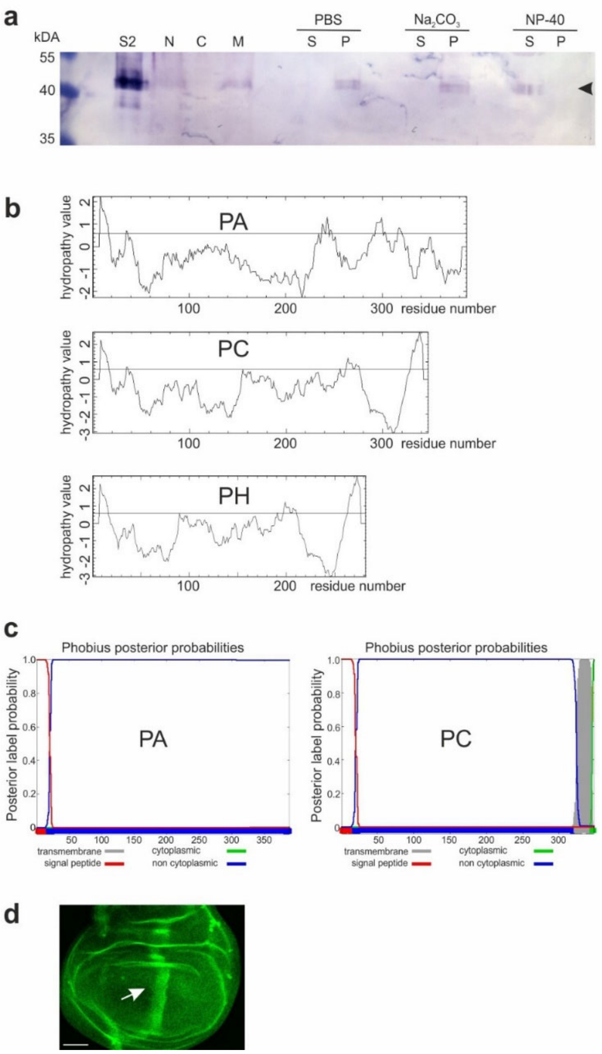

Figure S1. Mnr encodes two classes of membrane-bound proteins (**a**) Mnr protein in S2 cells is localized in the membrane fraction (M), not in the cytosol (C), and little in the nuclear fraction (N). The latter may result from a co-purification with membranes from the ER. Neither washes with PBS or alkaline treatment with Na2CO3 cause solubilization; the Mnr protein is found in the pellet fraction (P), whereas with Nonidet P-40 (NP-40) Mnr is solubilized (S). (**b**) In silico prediction of hydropathy value of proteins PA, PC and PH with EMBOSS using Kyte & Doolittle hydropathy parameters. (**c**) In silico predictionfor signal peptide and transmembrane domains according to Phobius for proteins PA and PC. (**d**) Induction of Mnr expression along the antero-posterior axis (arrow) in wing imaginaldiscs detected by anti-Mnr antibody staining. Genotype: EP555/+; ptc-Gal4 /+. Size bar, 50 µm.

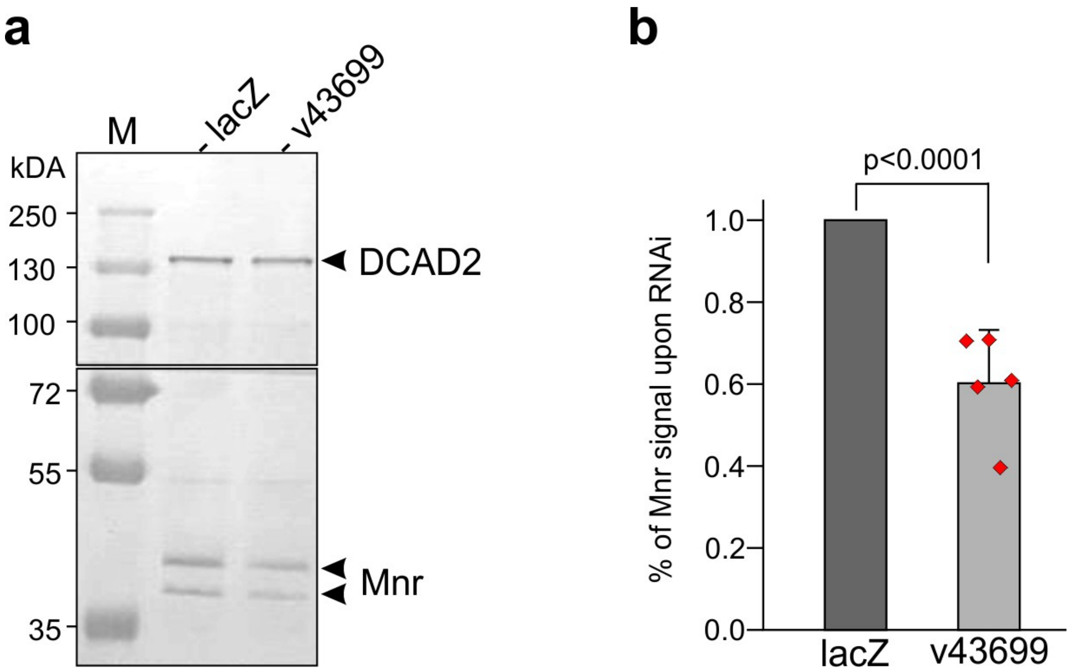

Figure S2. RNAi mediated knockdown of *mnr* expression by v43699 overexpression in the eye (**a**) Representative Western blot with protein extracts from female heads. In the control (lacZ), UAS-lacZ was overexpressed in the developing eye with gmr-Gal4; whereas RNAi against *mnr* was induced by likewise v43699 overexpression. The blot was cut to detect *Drosophila* E-cadherin (DCAD2) at roughly 130 kDA (arrow) and Mnr protein (arrowheads) simultaneously. Size standard (M), apparent protein weight is indicated in kDa. (**b**) Protein amount was determined relative to *Drosophila* E-cadherin from five blots derived from two biologically replicates using Image J *gels* analysis tool; statistical evaluation shows highly significant reduction down to about 60% upon RNA interference. The extracts contained Mnrprotein from the entire head, including the brain, whereas the knock down was specific to the developing eye, perhaps explaining the limited effectivity.

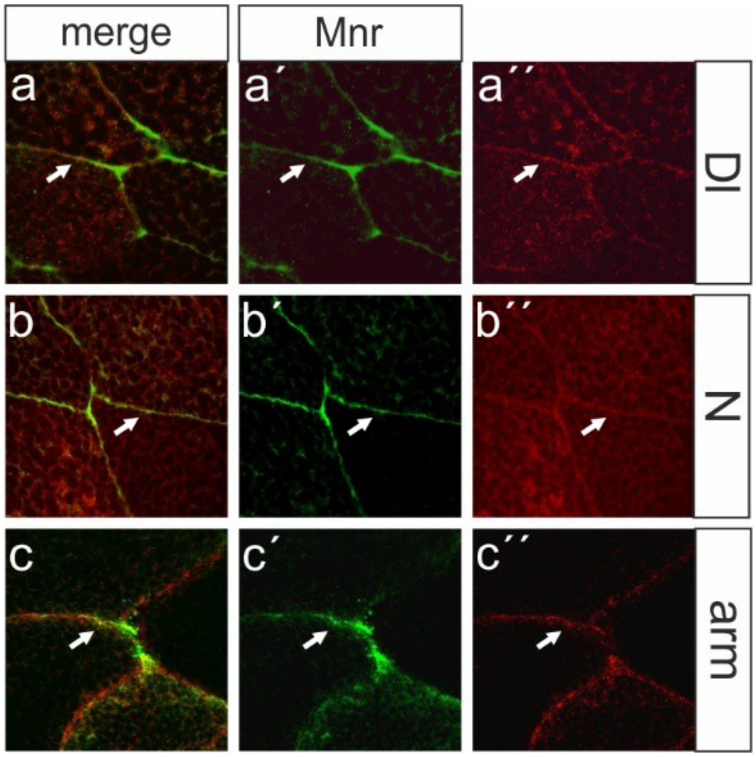

Figure S3. Co-localization of Mnr protein with Notch and Delta Co-localization of Mnr protein (green) with Delta (Dl) (**a**–**a′′**), Notch (N) (**b**–**b′′**) and Armadillo (arm) (**c**–**c′′**) in salivary gland cells (each in red). Arrows point to membranes that show co-localization. Overlap in the merge (**a**–**c**) appears yellow.

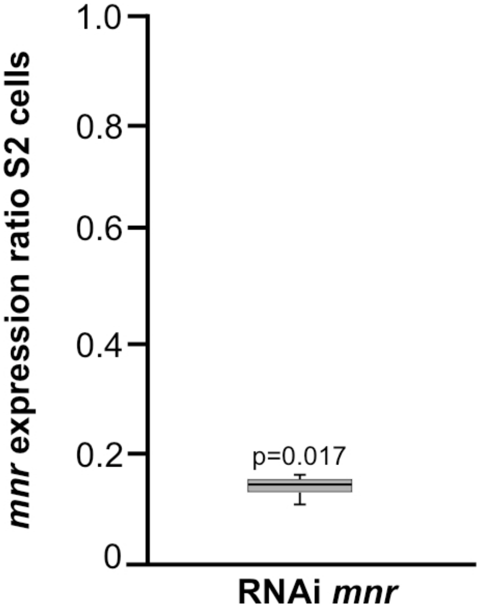

Figure S4. RNAi mediated knockdown of *mnr* expression in S2 cells Quantification of *mnr* expression levels in S2 cells subjected to RNAi mediated knockdown of RA and RC transcripts; qRT-PCR was performed with primers in exon 7 detecting all transcripts. Reference genes *cyp33* and *tbp*, respectively, were chosen based on variance and Cq value. Expression was compared to untreated cells; *mnr* RNAi resulted in more than 85% downregulation (0.143 fold expression in treated versus untreated cells). Data were obtainedfrom three biological and two technical replicates. Mini-max depicts 95% confidence, mediancorresponds to expression ratio. P-value is given above the bar. PFRR from REST ($p<0.05$) was used to test significance.
